# Susceptibility to myocardial ischemia reperfusion injury at early stage of type 1 diabetes in rats

**DOI:** 10.1186/1475-2840-12-133

**Published:** 2013-09-17

**Authors:** Haobo Li, Zipeng Liu, Junwen Wang, Gordon T Wong, Chi-Wai Cheung, Liangqing Zhang, Can Chen, Zhengyuan Xia, Michael G Irwin

**Affiliations:** 1 Department of Anesthesiology, The University of Hong Kong, Hong Kong SAR, China; 2 Department of Biochemistry, The University of Hong Kong, Hong Kong SAR, China; 3 Shenzhen Institute of Research & Innovation, The University of Hong Kong, Shenzhen, China; 4 Affiliated Hospital of Guangdong Medical College, Guangdong, China; 5 Department of Cardiology, Affiliated Hospital of Guangdong Medical College, Guangdong, China

**Keywords:** Ischemia reperfusion injury, Diabetes mellitus, Infarct size

## Abstract

**Background:**

Large body of evidences accumulated in clinical and epidemiological studies indicate that hearts of diabetic subjects are more sensitive to ischemia reperfusion injury (IRI), which results in a higher rate of mortality at post-operation than that of non-diabetes. However, experimental results are equivocal and point to either increased or decreased susceptibility of the diabetic hearts to IRI, especially at the early stage of the disease. The present study was designed to test the hypothesis that the duration/severity of the indexed ischemia is a major determinant of the vulnerability to myocardial IRI at early stage of diabetes.

**Methods:**

Four weeks streptozotocin (STZ)-induced diabetic (D) and non-diabetic (C) Sprague–Dawley rats were randomly assigned to receive 30 or 45 min of left anterior descending artery ligation followed by 2 or 3 hours of reperfusion, respectively. Cardiac function was recorded by using Pressure-Volume (PV) conduction system. Myocardial infarct size was determined with triphenyltetrazolium chloride staining. Plasma Creatine kinase-MB (CK-MB), Lactate dehydrogenase (LDH) release, myocardial nitric oxide(NO) content and nitrotyrosine formation, 15-F_2t_-Isoprostane and plasma superoxide dismutase (SOD) were measured with colorimetric assays. Cardiomyocyte apoptosis was assessed by TUNEL staining. Myocardial TNFα, Caspase-3, STAT3, Akt, and GSK-3β were determined by Western blotting.

**Results:**

Prolongation of ischemia but not reperfusion from 30 min to 45 min significantly increased infarct size in D compared to C rats (*P* < 0.05), accompanied with significantly increased plasma CK-MB (*P* < 0.05). Prolongation of the duration of either ischemia or reperfusion significantly increased plasma LDH release and myocardial 15-F_2t_-Isoprostane and reduced plasma SOD activity, with concomitant reduction of myocardial NO and increase of nitrotyrosine formation in D relative to C (*P* < 0.05). Prolongation of ischemia and reperfusion significantly reduced left ventricular ejection fraction and increased the peak rate of pressure, accompanied with increased end systolic pressure in D relative to C rats (*P* < 0.05) but reduced phosphorylations of myocardial STAT3 at site Ser727 and Akt at site Ser473 as well as GSK-3β at Ser 9 (*P* < 0.05).

**Conclusions:**

Diabetic hearts, even at early stage of the disease are more sensitive to IRI, and this increased severity of post-ischemic myocardial injury depends more on the duration of ischemia than that of reperfusion.

## Background

Despite of the current optimal therapy, the mortality and morbidityof patients with ischemic heart disease (IHD) remains high [1], particularly in patients with diabetes mellitus (DM) [2]. Myocardial infarct size is a major determinant of prognosis in patients with IHD, and development of a novel strategy to limit infarction is of great clinical importance. Clinical studies showed that DM increased the susceptibility of myocardium to ischemia-reperfusion injury (IRI) [3,4]. Larger infarct size, higher rates of new onset of severe congestive heart failure after reperfusion therapy were observed in DM patients than in non-DM patients [4]. In consistent with the change in myocardial vulnerability to infarction, patients with diabetes exhibit worse recovery after acute myocardial infarction (AMI) evidenced as worse short- and long-term prognosis after AMI [5,6]. Of particular relevance, studies showed that cardiovascular event risk in DM patients without prior myocardial infarction was comparable to the risk in non-diabetic patients with prior myocardial infarction [2]. Diabetes not only renders the heart more vulnerable to ischemic insult but also abolishes or compromises the effectiveness of cardioprotective interventions such as ischemic pre- or post-conditioning [7,8]. These available clinical evidences [7,8] strongly support the notion that DM increases the sensitivity of the heart to IRI.

Despite the overwhelming clinical data indicating that the diabetic heart is more sensitive to IRI, results from experimental diabetic animal models studies were contradictory. Some studies have shown that the diabetic heart is less sensitive to ischemic injury [7,9,10], while others have indicated that the diabetic heart is more sensitive [8,11,12]. Others found no differences in the vulnerability of the diabetic heart to IRI relative to non-diabetes [13,14]. Several factors have been proposed to explain the discrepancy in the effects of DM on the vulnerability to myocardial IRI in animal studies, which includes the duration and the severity of the diabetic state, experimental protocols (e.g. degree of ischemia and reperfusion, type of animal species), and metabolic profiles after diabetic induction. Among them, inconsistency of experimental protocols has been suggested to be the main cause. Marfella et. al [15] reported that myocardial infarct size after 25 minutes(min) ischemia followed by 2 hours reperfusion was larger in streptozotocin (STZ)-induced diabetic rats as early as 8 days after the induction of diabetes than that in non-diabetic rats. However, reduction of infarct size was observed in 2 weeks STZ-induced diabetic rats with 30 min ischemia/2 hours reperfusion [16]. Due to the fact that different protocols and animal species were employed in different studies, results gained from different studies regarding the vulnerability of the diabetic heart at the early stage of the disease, are not comparable, as such it remains unclear whether or not the diabetic hearts at the early stage of diabetes are more sensitive to IRI. Therefore, a systemic study by applying multiple durations of ischemia and/or reperfusion at the same diabetic model is needed to address this question.

Excess oxidative stress caused by the burst of reactive oxygen species (ROS) in the presence of inadequate antioxidant defense, has been considered as a potential mechanism governing the increased sensitivity of the diabetic heart to IRI [17]. Under physiological condition, in which ROS are in low concentrations, it serves as signaling molecules which is beneficial to the heart [18]. However, under pathological (including diabetes) condition, ROS elicits harmful effects when produced in excess [19]. ROS production is increased both during ischemia and within a few minutes of reperfusion, which leads to and exacerbates myocardial IRI.

To our knowledge, there is no systematic study to address the impact of durations of ischemia and/or reperfusion on the sensitivity of diabetic heart to IRI, especially at the early stage of the disease. Therefore, the aims of this study were to employ different durations of myocardial ischemia and reperfusion in STZ-induced diabetic rats at the early stage of the disease (4 weeks) to address whether or not diabetic heart is more sensitivity to IRI.

## Methods

### Induction of diabetes

Male Sprague-Dawley rats (250 ± 10 g, 6–8 weeks) obtained from the Laboratory Animal Service Center (the University of Hong Kong) were used in this study. All rats were housed and given free access to standard rat chow and water in accordance with the principles of Animal Care of the University of Hong Kong. The experimental protocol used in this study was approved by the Committee on the Use of Live Animals in Teaching and Research (CULATR). Diabetes was induced by a single tail vein injection of STZ at the dose of 65 mg/kg bodyweight (Sigma-Aldrich, St. Louis, MO) in 0.1 M citrate buffer (pH 4.5) or citrate buffer alone as control under anesthesia with a combination of ketamine 67.7 mg/kg bodyweight and xylazine 6.77 mg/kg bodyweight. After 72 hours injection, blood glucose was measured using a One Touch Ultra Glucose meter (Life Scan Inc. USA) and rats with blood glucose levels over 15 mM were considered diabetes.

### Measurements of general characteristics

During treatment period, water intake was assessed daily; food consumption and body weight were monitored weekly. Blood glucose levels (mM) were measured once a week by using a One Touch Ultra Glucose Meter (Life Scan Inc. USA). At termination (4 weeks after the onset of diabetes at the absent or present of myocardial ischemia reperfusion), rats were weighed and then euthanized following anaesthesia with an intraperitoneal injection of pentobarbital sodium (65 mg/kg). Blood samples were obtained from carotid artery after an overnight fast of 8–10 h, and plasma was extracted and stored at −80°C until analyzed. Serum Total Cholesterol (TC) and Triglyceride (TG) were determined using commercially available kits (Stanbio laboratory, TX, USA), respectively.

### Experimental protocol

After completion of instrumentation and surgery, rats were allowed a 15 min equilibration period. Diabetic (D) and non-diabetic (C) rats were randomly assigned to receive 30 or 45 min of left anterior descending (LAD) artery occlusion followed by either 120 or 180 min of reperfusion, respectively.

### Myocardial ischemia reperfusion injury *in vivo*

Rats were anesthetized by intraperitoneal injection of sodium pentobarbital (65 mg/kg) and the trachea was cannulated with a polyethylene tube connected to a rodent respirator (Harvard Apparatus, Holliston, MA) with a tidal volume of 1.0 mL/100 mg body weight (60 breaths/min) [20]. Then left thoracotomy was performed between the fourth and fifth ribs. The pericardial tissue was removed under a microscope and the LAD artery was ligated with 6–0 silk suture using a snare occluder [21]. The rats were then subjected to 30 min or 45 min of LAD ligation followed by 120 min or 180 min of reperfusion.

### Measurement of left ventricular function

The global cardiac functions under the load dependent and independent conditions were monitored by using a Pressure-Volume (PV) conductance catheter (AD Instruments, Colorado Springs, CO, USA) placed into the left ventricle through right carotid artery and connected to a computer equiped with an Advantage PV control box software (AD Instruments, Colorado Springs, CO, USA). Briefly, rats were anesthetized with an intraperitoneal injection of sodium pentobarbital (65 mg/kg) and ventilated with room air mixed with pure oxygen using a rodent ventilator. A Millar ultra-miniature PV catheter was advanced into the left ventricle (LV) of rat beating heart. The hemodynamic parameters in each rat were measured in the chest opened rats and the LAD was occluded after stabilization for 10 min. At baseline and at the end of reperfusion, the cardiac functions were recorded and compared between the C and D rats. The cardiac functional parameters recorded include heart rate (HR); left ventricular end-systolic pressure (LVESP); left ventricular end-diastolic pressure (LVEDP); stroke volume (SV); left ventricular ejection fraction (LVEF); stroke work (SW). The load-independent contractility parameters including the peak rate of pressure increase (dP/dt max); arterial elastance (Ea = LVESP/SV); the peak rate of pressure decrease (dP/dt min); the relaxation time constant calculated by Weiss method (Tau) were analyzed using Labchart 7 software (AD Instruments, Colorado Springs, CO, USA).

### Determination of myocardial infarct size

At the end of the experiment, myocardial infarct size was measured using TTC (1% 2,3,5-triphenyltetrazolium chloride) staining as we described [21]. Briefly, the LAD was re-occluded and cannulated just distal to the occlusion site. Ten milliliters of saline and 10 mL of patent blue dye were injected at equal pressure into the LAD and left atrium, respectively, to delineate the anatomic area at risk (AAR) subjected to prolonged occlusion and reperfusion and the non-ischemic normal zone. The heart was immediately fibrillated, removed, and sliced into serial transverse sections 6 to 7 mm in width. The unstained AAR was separated from the blue stained normal area, and the two regions were incubated at 37°C for 20 to 30 min in 1% TTC in 0.1 mol/L phosphate buffer adjusted to pH 7.4, and photographed with a digital camera. TTC stains non-infarcted myocardium a brick red color because of the presence of a formalin precipitate, resulting from reduction of TTC by dehydrogenase enzymes present in viable tissue. Infarcted myocardium remains unstained. Infarcted and non-infarcted myocardium within the AAR were digitally measured using an image analysis software (ImageJ, version 1.47, National Institutes of Health, Bethesda, MD). Infarct size (IS) was expressed as a percentage of the AAR.

### Measurements of plasma Creatine kinase-MB (CK-MB) and Lactate dehydrogenase (LDH) levels

Plasma CK-MB levels were determined using a commercially available rat ELISA kit (R&D Systems, Minneapolis, MN). Plasma LDH levels were measured using a commercially available kit (Roche, Germany).

### Determination of plasma free 15-F_2t_-Isoprostane (15-F_2t_-IsoP) and superoxide dismutase (SOD) activity

15-F_2t_-IsoP, a specific marker of oxidative stress *in vivo*, was measured using an enzyme immunoassay kit (Cayman Chemical, MI, USA) as described previously [21]. Plasma samples homogenates in PBS were purified using Affinity Column and Affinity Sorbent (Cayman Chemical, MI, USA). The absorbance from the enzymatic reaction was detected at 412 nm. The plasma 15-F_2t_-isoP levels are expressed as pg/ml. The total SOD activity in plasma was assayed using a reagent kit (Cayman Chemical, MI, USA) according to the manufacturer’s recommendations.

### Determination of myocardial levels of nitric oxide and nitrotyrosine

Concentrations of nitrites (NO^2−^) and nitrates (NO^3−^), the stable end products of nitric oxide (NO), were determined at the end of reperfusion in the ventricular tissue by a NO colorimetric assay kit (BioVision, Inc. California). The values of cardiac NO production were expressed as the total nitrate and nitrite levels. Cardiac nitrotyrosine, a footprint of peroxynitrite was measured with chemiluminescence detection (Millipore, Billerica, MA, USA) according to the manufactory’s instruction.

### Protein extraction and immunoblotting

Heart tissue were homogenized using lysis buffer [(20 mmol/L Tris–HCl pH = 7.5, 50 mmol/L 2-mercaptoethanol, 5 mmol/L EGTA, 2 mmol/L EDTA, 1% NP40, 0.1% sodium dodecyl sulfonate (SDS), 0.5% deoxycholic acid, 10 mmol/L NaF, 1 mmol/L PMSF, 25 mg/mL leupeptin, 2 mg/mL aprotinin)], sonicated and centrifuged at 12 000 g for 20 min at 4°C. Protein concentrations were determined using the Bradford assay (Bio-Rad, USA). Samples containing equal amounts of proteins were separated on a 10% SDS-polyacrylamide gel and then the proteins were transferred to PVDF membrane. Membranes were blocked with 5% non-fat milk in Tris-Buffered Saline (TBS)-Tween and were incubated with primary antibodies overnight at 4°C at the following dilutions: Akt, signal transducer and activator of transcription 3 (STAT3), glycogen synthase kinase-3 beta (GSK-3β) and GAPDH (Cell Signaling Technology, Beverly, MA) 1:1000; phosphor-STAT3 (Ser 727), phosphor-Akt (Ser 473) and phosphor-GSK-3β (Ser 9) (Cell Signaling Technology, Beverly, MA) 1:500. After washing with phosphate buffered saline-tween (PBST), membrane strips were washed and incubated with horseradish peroxidase (HRP)-conjugated anti-rabbit and detected by enzymatic chemiluminescence.

### Immunohistochemistry (TUNEL)

Paraffin-embedded left ventricular tissue blocks were sectioned at 5 mm. Then, the sections were dewaxed and rehydrated. Slides were incubated in 3% hydrogen peroxide/methanol. Antigen retrieval was performed by heating in 10 mM sodium citrate buffer for 10 min. Sections were incubated in anti-collagen I antibody (abcam, USA) at 1:50 dilution for 12 h at 4°C. 3,3′-Diaminobenzidine Substrate Chromogen System (Dako, S1699) was employed in the detection procedure. Subsequently, the sections were counterstained with nuclear fast red for 3 min. Finally, the sections were dehydrated in ethanol, cleared in xylene, mounted and observed in a light microscope. The sections were observed in the light microscope by an investigator who was initially blinded to treatment groups, and five randomly selected fields of each slide were semi-quantified and averaged using the software Image J 1.42 (National Institutes of Health) according to its instructions.

### Statistical analysis

All the values are expressed as mean ± standard error of the mean (S.E.M). One-way analysis of variance (ANOVA) was used for statistical analyses (GraphPad Prism, USA) of data obtained within the same group of rats and between groups of rats, respectively, followed by Tukey’s test for multiple comparisons of group means. *P* < 0.05 was considered statistically significant.

## Results

### General characteristics at termination

STZ-induced diabetic rats displayed hyperglycemia, hyperlipidemia, polydipsia and polyphagia evidenced as significantly increased plasma glucose, triglycerides, cholesterol, water intake and food consumption, compared with age-matched controls (all *P* < 0.05, Table 1). The body weight in the diabetic group was lower than that in the control group (*P* < 0.05), while the heart/body weight ratio in the diabetic group were higher than that in the control group (*P* < 0.05).

**Table 1:** General characteristics before myocardial ischemia reperfusion

**Parameters**	**C**	**D**
Water intake (ml/kg/day)	134.7 ± 8.1	725.3 ± 9.3*
Food consumption (g/kg/day)	61.9 ± 3.1	164.7 ± 11.2*
Body weight (g)	478.2 ± 10.3	351.4 ± 8.9*
Plasma glucose (mM)	7.1 ± 0.5	28.9 ± 1.2*
Heart/body weight ratio (g/kg)	2.6 ± 0.1	3.0 ± 0.1*
Triglycerides (mg/dL)	126.09 ± 11.4	220.95 ± 9.7*
Cholesterol (mg/dL)	75.66 ± 6.7	89.70 ± 9.1

All values are expressed as mean ± S.E.M. n = 7 per group. Water intake and food consumption were the average value of four weeks. Body weight, Heart/Body weight ratio, plasma glucose, triglycerides and cholesterol were measured at 4-week after STZ injection in these two groups. All of the indices were measured between groups before inducing myocardial ischemia reperfusion. *P < 0.05 vs. C.

### Infarct size and myocardial injury after IR

**Figure 1:**
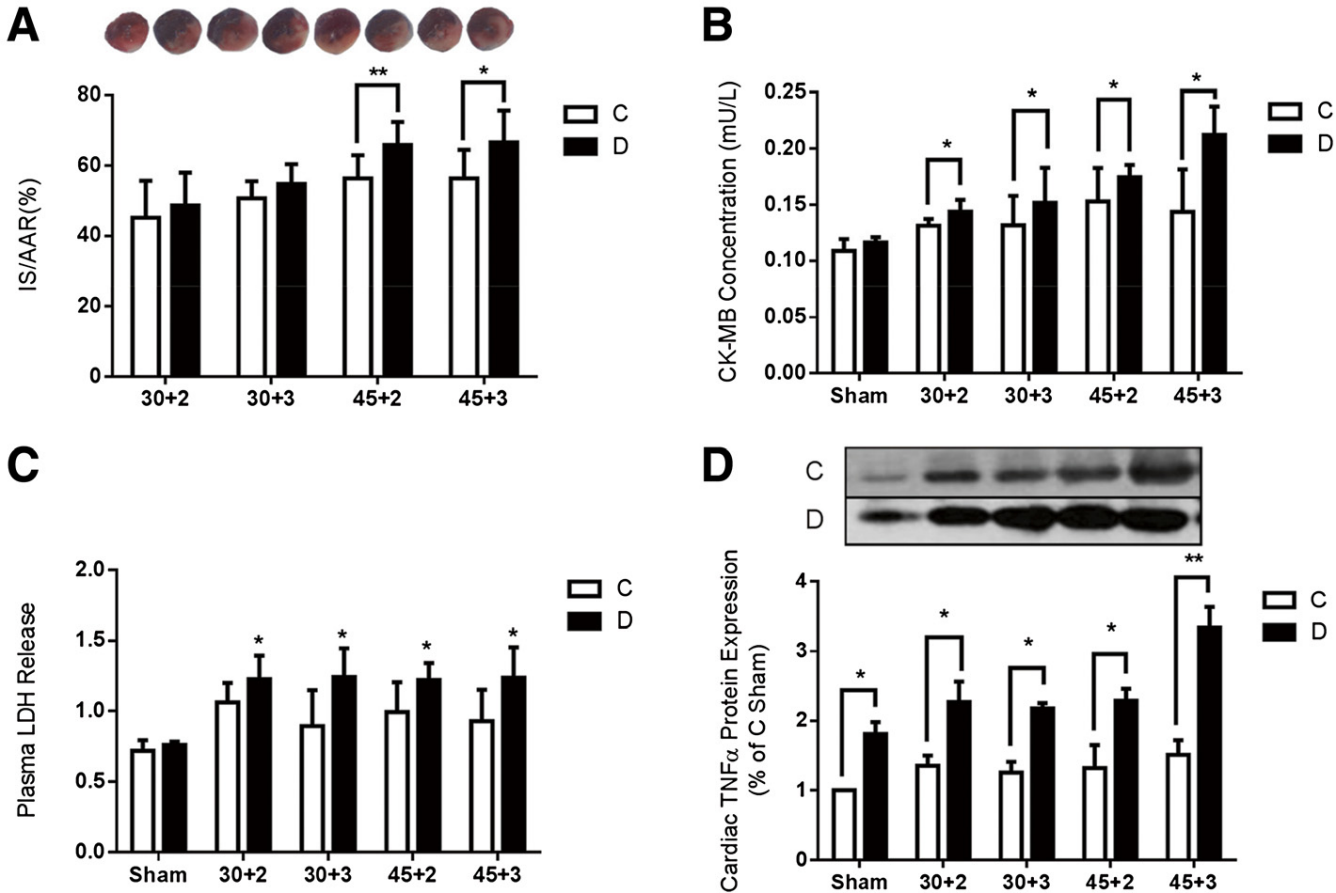
**Myocardial infarct size and cellular injury after myocardial ischemia reperfusion in control and diabetic rats. A**. Percentage of infarct size (IS) expressed as a percentage of the area at risk (AAR), **B**. Plasma CK-MB secretion assessed by ELISA kit, **C**. Plasma lactate dehydrogenase (LDH) leakage, **D**. Protein expression of cardiac Tumor necrosis factor α(TNF α). Data are expressed as mean ± SEM (n = 7 per group). *P < 0.05, **P < 0.01 in C vs. D.

**Figure 2:**
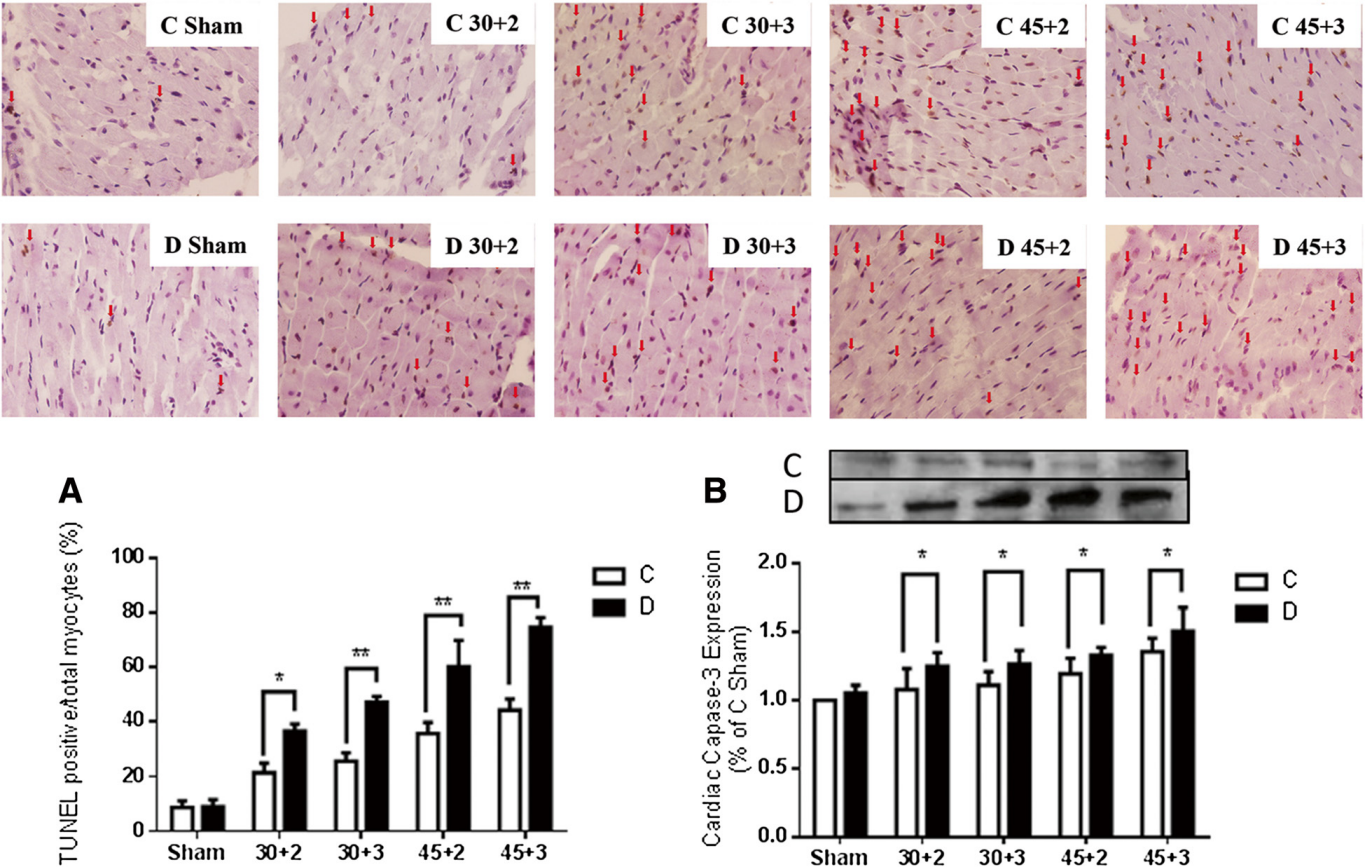
**Myocardial cell apoptosis assessed by TUNEL staining and cardiac caspase-3 protein expression before and after myocardial ischemia reperfusion. A**. Myocardial cell apoptosis assessed by TUNEL, TUNEL positive cells were stained brown as indicated by arrow, **B**. Cardiac caspase-3 protein expression. Data are expressed as mean ± SEM (n = 7 per group). *P < 0.05, **P < 0.01 vs. C.

As shown in Figure 1, myocardial infarct size (IS) was bigger in D than that in C but could not reach statistical significance when the rats were subjected to 30 min of LAD occlusion followed by either 2 or 3 hours. However, when the duration of LAD occlusion was extended to 45 min followed by either 2 or 3 hours, significant bigger infarct size was observed in D relative to C rats (Figure 1A) (*P* < 0.05). Similar to our previous report [12,21], at the end of 30 min LAD with 2 or 3 hours reperfusion, the myocardial IS between control and diabetic rats did not reach statistical significance, while the release of plasma CK-MB was significantly increased in D relative to C after reperfusion (Figure 1B) (*P* < 0.05), which was consistent with a significant increase of plasma LDH (*P* < 0.05) and cardiac TNFα protein expression (*P* < 0.05) after 30 min LAD occlusion with 2 or 3 hours reperfusion (Figure 1C and D). Diabetic rats subjected to 45 min LAD occlusion with either 2 or 3 hours reperfusion resulted in a significant increase in IS when compared to that of C rats (Figure 1A). Of note, when we extended the duration of LAD to 60 min, we observed that the majority of the rats in both the C and the D groups could not survive (data not shown). As shown in Figure 2A, after IR, apoptotic myocytes was significantly increased in diabetic rats evidenced as larger number of TUNEL-staining positive cells compared with controls (Figure 2A) (*P* < 0.05), which was coincident with a significantly increase of cardiac caspase-3 protein expression after IR (Figure 2B) (*P* < 0.05).

### Left ventricular functions

**Figure 3:**
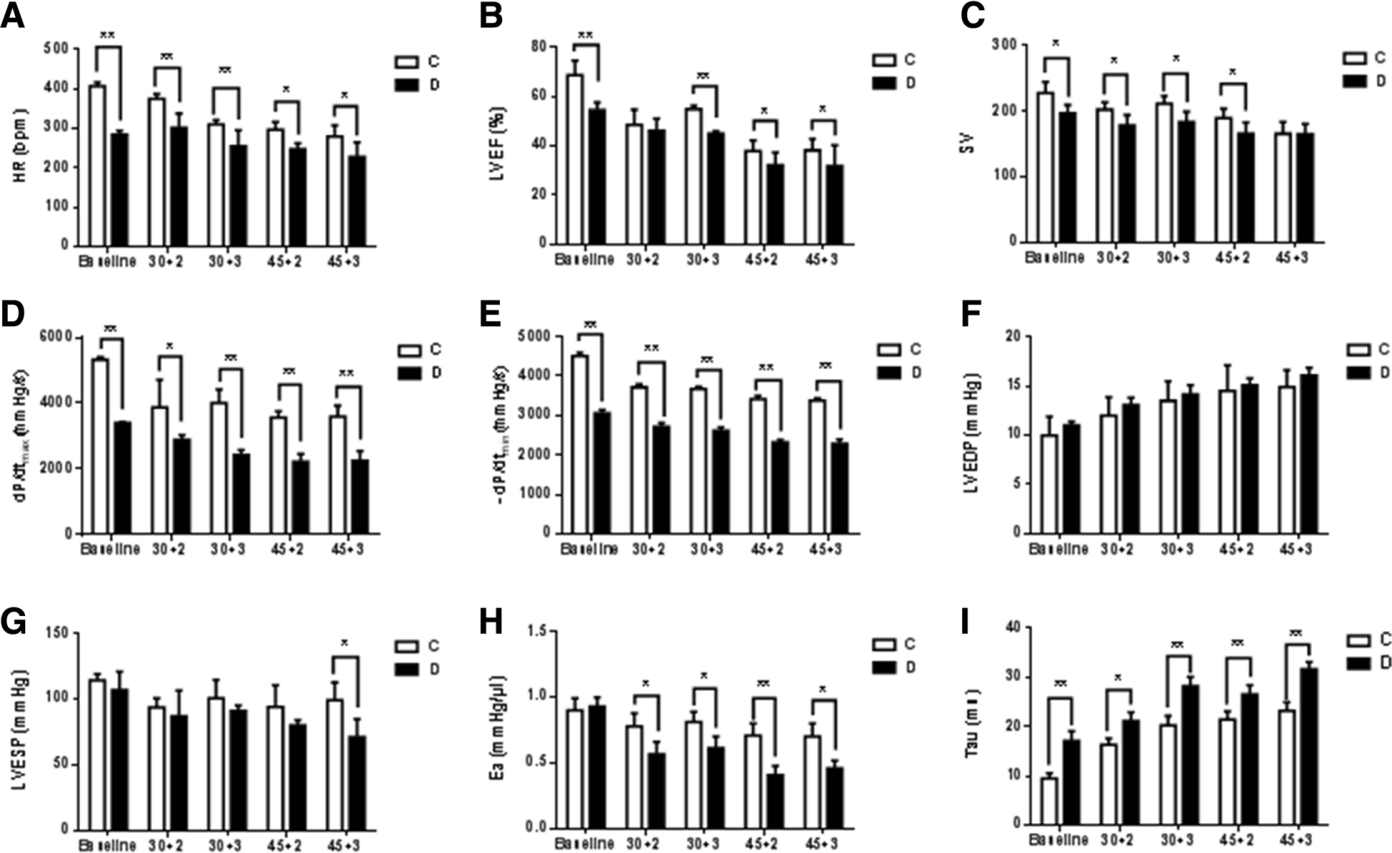
**Left ventricular function before and after ischemia reperfusion in control and diabetic rats. A**. HR: heart rate, **B**. LVEF: left ventricular ejection fraction, **C**. SV: stroke volume, **D**. dP/dt max: load-independent contractility parameters including the peak rate of pressure increase, **E**. dP/dt min: the peak rate of pressure decrease, **F**. LVEDP: left ventricular end-diastolic pressure, **G**. LVESP: left ventricular end-systolic pressure, **H**. Ea: arterial elastance (Ea = LVESP/SV), **I**. Tau: relaxation time constant calculated by Weiss method. Data are expressed as mean ± SEM (n = 7 per group). *P < 0.05, **P < 0.01 vs. C.

Before inducing IR (baseline) and at the end of IRI, cardiac function was determined by using a pressure volume conduction system. As shown in Figure 3, STZ-induced diabetes displayed poor cardiac function evidenced as significantly lower LVEF, SV, dP/dt max, and dP/dt min compared to controls (Figure 3B, D, and E) (*P* < 0.01 vs. C). Diabetes rats displayed a significant decrease in HR before (baseline) and after IRI (Figure 3A) (*P* < 0.01 vs. C). LVEF, a surrogate marker of heart failure status was significantly reduced in diabetic rats relative to control rats when the rats were subjected to 30 min ischemia followed by 3 hours but not by 2 hours reperfusion. When the duration of ischemia was 45 min, LVEF in the diabetic group was constantly lower than that in the control group following either 2 or 3 hours of reperfusion (Figure 3B) (*P* < 0.05 vs. C). At baseline and the end of IRI, the important contractile function index dP/dt_max_ and dP/dt_min_ were significantly decreased in diabetic rats relative to controls (Figure 3D and E) (*P* < 0.01 vs. C), despite that no significant difference was observed in LVEDP (Figure 3F). Significant decrease in LVESP was only observed in diabetic rats subjected to 45 min LAD occlusion followed by 3 hours reperfusion (Figure 3G) (*P* < 0.05 vs. C). The value of Ea did not significantly differ between diabetic and control groups at baseline, but it was significantly reduced in diabetic rats relative to controls when the rats were subjected to either 30 min or 45 min of reperfusion followed by 2 or 3 hours of reperfusion (Figure 3H) (*P* < 0.01 vs. C). Tau Weiss was significantly increased in diabetic rats as compared to control rats (Figure 3I) (*P* < 0.01).

### Oxidative stress and antioxidant status after inducing IR

**Figure 4:**
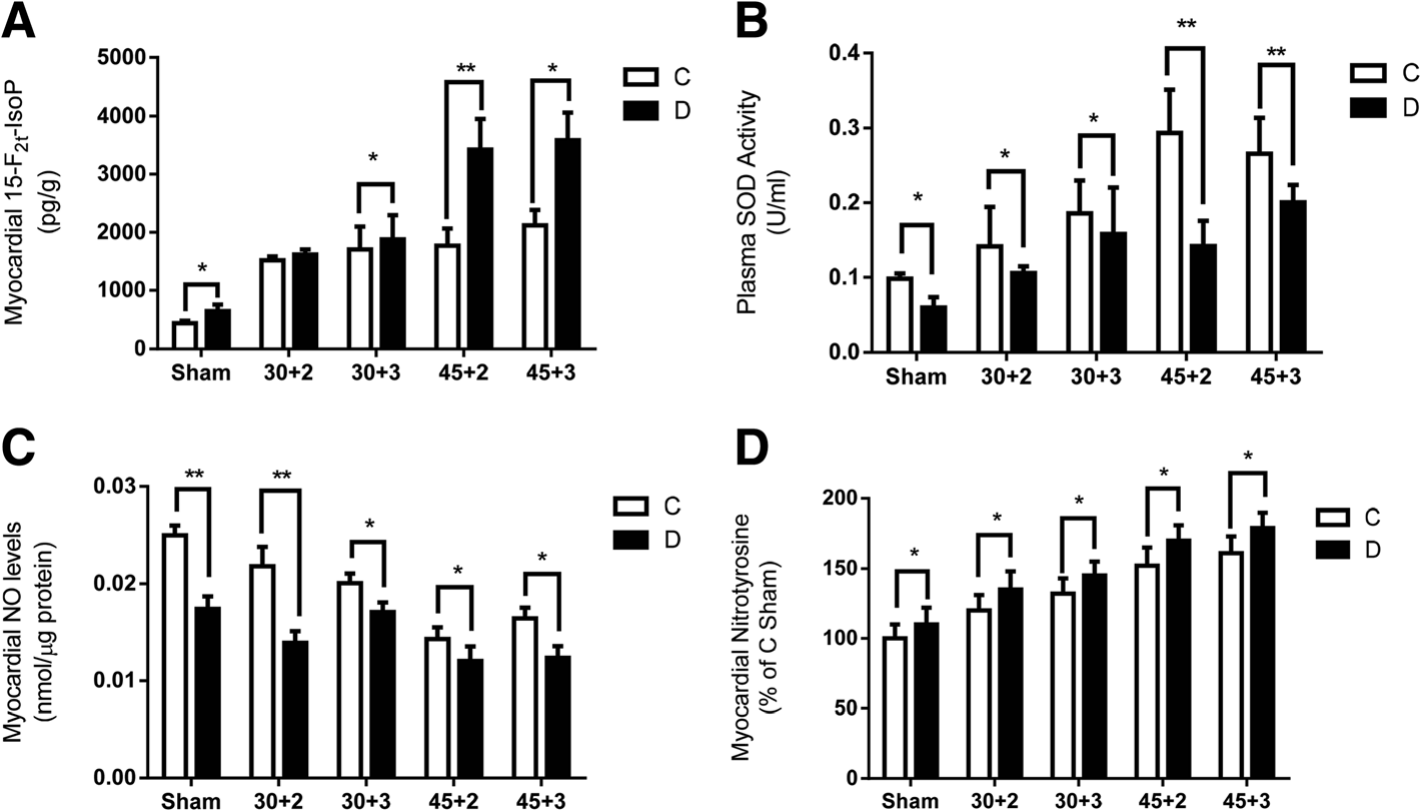
**Biochemical assessment before and after ischemia reperfusion in control and diabetic rats. A**. Myocardial levels of 15-F_2t_-Isoprostane, **B**. Plasma levels of SOD, **C**. Cardiac NO production, **D**. Myocardial nitrotyrosine. Data are expressed as mean ± SEM (n = 7 per group). *P < 0.05, **P < 0.01 vs. C.

As shown in Figure 4, after IR, plasma 15-F_2t_-IsoP was elevated in both the control and the diabetic rats (*P* < 0.05), accompanied with decreased plasma SOD (*P* < 0.05) (Figure 4A and B). After 30 min LAD occlusion with 2 hours reperfusion, plasma 15-F_2t_-IsoP in diabetic group was relatively higher than that in the control group but the difference did not reach statistical significance, while when the duration of reperfusion was extended to 3 hours or the duration of LAD occlusion extended to 45 min, plasma 15-F_2t_-IsoP was significantly increased in diabetic compared with control group (Figure 4A) (*P* < 0.05). After 4 weeks of diabetes, baseline (before inducing IR) myocardial nitric oxide production in diabetic rats was lower than that in control rats (*P* < 0.05). After 30 min LAD occlusion/2 hours reperfusion or 45 min LAD occlusion followed by either 2 hours or 3 hours reperfusion, myocardial nitric oxide was significantly increased in diabetic rats relative to controls (Figure 4C) (*P* < 0.05), accompanied with a significant increase myocardial nitrotyrosine formation (Figure 4D) (*P* < 0.05).

### Myocardial STAT3, Akt, and GSK-3β expression after IR

**Figure 5:**
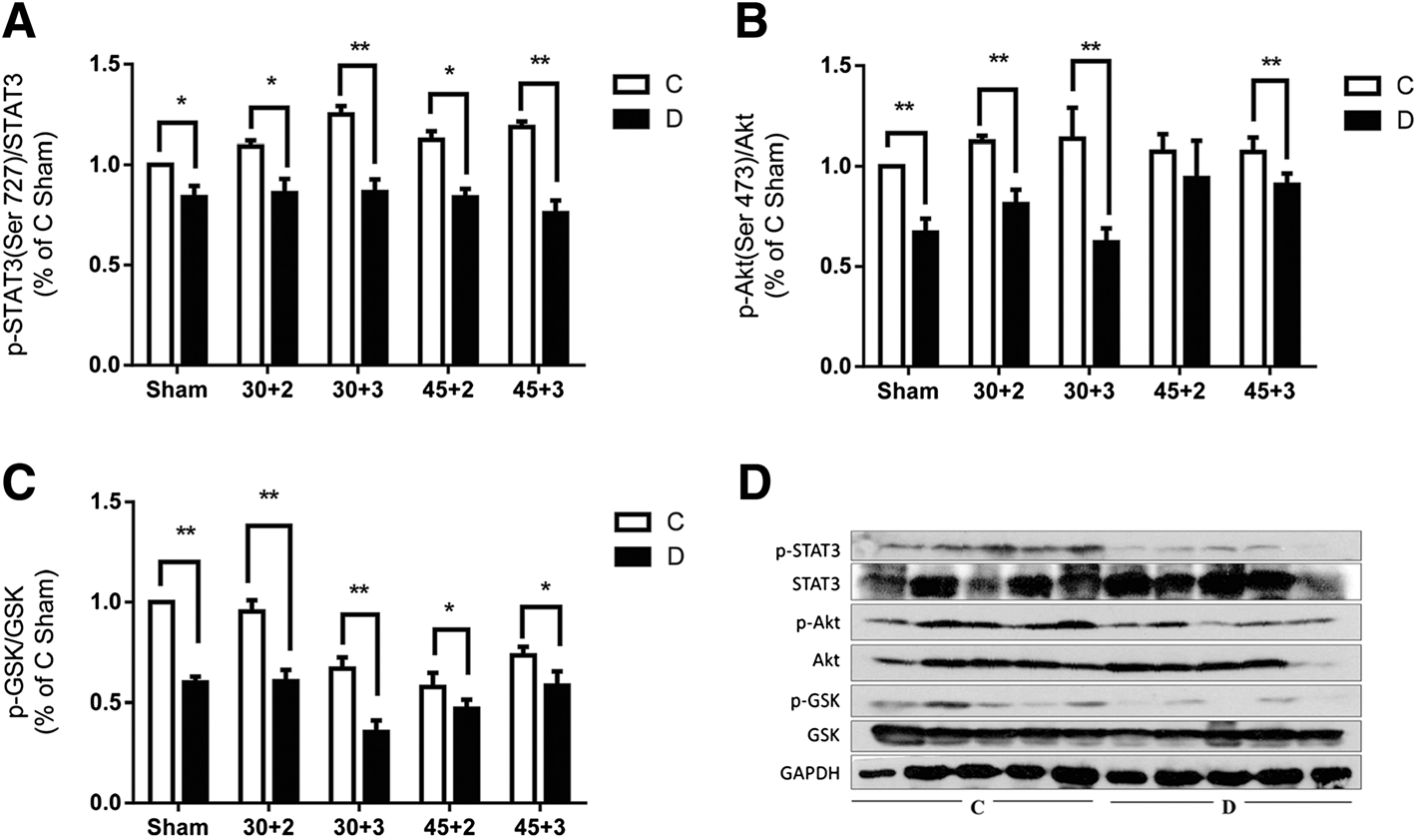
**Myocardial STAT3, Akt and GSK-3β protein expression and their phosphorylation status before and after ischemia reperfusion in control and diabetic rats. A**. Total and phosphorylation (Ser727) of STAT3, **B**. Total and phosphorylation (Ser473) of Akt, **C**. Total and phosphorylation (Ser9) of GSK-3β, **D**. Representative Western blots. Mean band density was normalized relative to GAPDH. Data are expressed as mean ± SEM (n = 7 per group), *P < 0.05, **P < 0.01 vs. C.

Studies from ours [12,21] and others [22] have shown that phosphoinositide 3-kinase (PI3K)/Akt and Janus kinase 2 (Jak2)/STAT3 signaling pathways may be involved in antioxidant-mediated NO production and subsequently contribute to myocardial protection against ischemia reperfusion injury. As shown in Figure 5B, before and after inducing IR, phosphorylation of myocardial Akt at Site Ser473 was significantly reduced in diabetic rats relative to controls (*P* < 0.05) (Figure 5B), and similarly, its downstream molecule, glycogen synthase kinase-3β (GSK-3β), was significantly reduced in diabetic rats compared to controls both before and after inducing IR as evidenced by reduced phosphorylation at Ser9 (Figure 5C) (*P* < 0.05). As we previously reported [21], STAT3 phosphorylation contributes to myocardial eNOS activation and NO production, therefore, we investigated the change in myocardial protein levels of STAT3. As showed in Figure 5A, a significant reduction of p-STAT3 was observed in diabetic rats compared to controls both before and after inducing IR (Figure 5A) (*P* < 0.05).

## Discussion

In this study, using *in vivo* ischemia reperfusion model incorperating with the application of different durations of ischemia and reperfusion in the same disease model, we demonstrated that the diabetic hearts, even at the early stage of the disease, are more sensitive to IRI than hearts from non-diabetic subjects. Furthermore, we showed that the severity of the post-ischemic myocardial injury of the diabetic hearts depended more on the duration of ischemia than that of reperfusion. The severity of oxidative stress and reduction of endogenous antioxidant capacity and the impairment of protective signaling pathway related to the activation of STAT3 and Akt are likely the major mechanisms responsible for the increased post-ischemic myocardial injury in diabetes after prolonged ischemic insult. To our knowledge, this is the first systematic study to investigate the mutual impact of duration of ischemia and reperfusion on myocardial tolerance to IRI in diabetes, particularly, in the early phase of diabetes. Our findings may help to resolve the discrepancy regarding whether or not hearts from diabetes at the early stage of the disease are less resistant to ischemic insult.

Although clinical studies have convincingly demonstrated that the diabetic heart is more vulnerable to IRI, results from experimental studies are still inconsistent, especially at the early stage of the disease. Smaller infarct size was observed after 30 min ischemia in diabetic rat hearts at 1 week after STZ injection than in controls, but this infarct size limiting effect was disappeared 8 weeks later [9]. Similarly, Ma et al. [10] reported that the diabetic hearts were more sensitive to IRI only at the late phase of the diabetes. However, enlargement of infarct size was observed as early as 8 days in STZ-induced diabetes [15]. Thus, despite of the consensus that hearts from chronic diabetic subjects are more sensitive to IRI than hearts from controls, results obtained from studies conducted at the early stage of diabetes are mostly inconsistent. Of note, there are numerous differences in experimental preparations and protocols in the reported studies, and a single factor cannot entirely explain the discrepancy in the effects of diabetes on the sensitivity of the heart to ischemia reperfusion injury. Therefore, in the present study, we applied different duration of ischemia and reperfusion in the same diabetic model to examine whether or not the diabetic heart is more vulnerable to IRI at its early stage. Studies in non-diabetes subjects have shown that a sigmoidal relationship exists between myocardial infarct size and duration of ischemia, where, 20 min of ischemia only induced extremely small infarct size [23,24], and significant impact of the duration of reperfusion only observed when ischemia time was shorter than 60 min in non-diabetes [24]. However, our previous study showed that myocardial infarct size in rats at 4 weeks of STZ-induced diabetes did not significantly differ from that in the non-diabetic rats when the rats were subjected to 30 min ischemia followed by 2 hours of reperfusion [21]. Therefore, in the current study we applied different durations of ischemia (30 min and 45 min) followed by either 2 hours or longer duration (3 hours) of reperfusion to see whether or not diabetic rats were more sensitive to IRI than non-diabetc rats. We found that enlargement of infarct size was only detectable when the diabetic rats were subjected to a longer enough (45 min) duration of ischemia with either 2 or 3 hours reperfusion, wherever the release of plasma CK-MB and plasma LDH were significantly increased in diabetic rats relative to controls after either 30 or 45 min of ischemia with 2 or 3 hours reperfusion. These indicate that myocardial cellular injury is more severe in diabetic than that in the control rats during reperfusion, despite that significant difference of infarct size was only observed when rats subjected to 45 min of ischemia with 2 or 3 hours reperfusion. Infarct size is considered as the gold standard in assessing the severity of heart damage after ischemia reperfusion, while CK-MB is regarded as the diagnostic hallmark for assessing post-ischemic myocardial infarction in patients after acute myocardial ischemia [25,26]. Reports from different studies showed that these two reliable parameters, infarct size and CK-MB, not always reach their peak values at the same time. In patients with acute myocardial ischemia, plasma CK-MB reaches its peak level at about ten hours after ischemia and predicts well with myocardial infarct size which reaches its peak level at five to seven days after reperfusion with thrombolytic therapy [26]. Similar findings have also been reported in a rat model, which showed that the increase in post-ischemic CK-MB peaked minutes after reperfusion while significant myocardial infarct size did not become apparent until after one hour of reperfusion [27]. In the present study, significant higher post-reperfusion CK-MB levels and LDH levels (a marker of cell necrosis) in diabetic rats correlated with more severely impaired post-reperfusion left ventricular function and increased numbers of apoptotic cardiomyocytes as compared to controls. In the current study, significant difference in post-reperfusion infarct size between diabetic and control rats were only detected when the rats were subjected to 45 minutes of ischemia with 2 or 3 hours of reperfusion but not after 30 min of ischemia followed by 2 hours reperfusion despite of the significant increase in CK-MB level and LDH levels. This indicates that the reperfusion time of 180 minutes may not be long enough to catch significant increase in infarct size in the diabetic group when the duration of ischemia was set at 30 min. In other words, enlargement of infarct size depended more on the duration of ischemia but not (or to a less degree) on that of reperfusion. In line with our present results, studies in dogs [28] and patients [29] showed that myocardial necrosis is time dependent and ischemia time serves as a determinant of infarct size and subsequent left ventricular dysfunction in individual patients with myocardial infarction. Significantly higher levels of CK-MB and LDH as well as more severe impairment in post-ischemic left ventricular function and increased apoptotic cardiomyocytes in the diabetic rats as shown in the present study indicate that myocardial injury is more severe in diabetic than in control rats, even at the early stage of the disease (4 weeks of diabetes). More importantly, results from current study also suggest that inconsistence of experimental protocols may be responsible for the discrepancy of the previously reported results.

Larger amount of studies support the concept that increased oxidative stress, such as a burst of reactive oxygen species production and reduction of antioxidant capacity contribute to myocardial tissue injury secondary to ischemia and reperfusion [30], especially in diabetic heart, in which hyperglycemia enhances myocardial oxidative stress and subsequently aggravates diabetic heart damage to IRI [31]. It has been widely accepted that prolongation of ischemia would dramatically increase myocardial pro-inflammatory cytokine TNFα production [32], which will subsequently increase the formation of nitrotyrosine and concomitantly reduce the activities of antioxidant enzymes, leading to a burst of ROS production which is detrimental to the myocardium [33]. As shown in the present study, STZ-induced diabetic rats exhibited increased myocardial oxidative stress manifested as increased myocardial 15-F_2t_-Isoprostane production and reduced plasma SOD activity, which was accompanied with increased myocardial TNFα protein expression, reduced myocardial nitric oxide production and increased myocardial nitrotyrosine formation. It has been documented that during ischemia and reperfusion, especially in the early few minutes of reperfusion, increased myocardial oxidative stress, such as augment of 15-F2t-IsoP production and reduction of SOD activity contribute to myocardial injury [27]. Similarly, in the present study, after ischemia reperfusion, diabetic rats displayed more severe oxidative stress, shown as significantly increased myocardial 15-F_2t_-IsoP production and reduction of plasma SOD activity relative to control. Of note, progressively significant increase of myocardial 15-F_2t_-IsoP production was observed when diabetic rats subjected to longer duration (from 30 min to 45 min) of ischemia but not that of reperfusion, which suggests that 15-F_2t_-IsoP production occurs mainly at the time of ischemia or in the early minutes of reperfusion and maintains at certain level when reperfusion sustained. This is consistent with our previous report showing that 15-F_2t_-IsoP increased at the time of reperfusion and progressively reduced during reperfusion and that 15-F_2t_-IsoP per se can exacerbate myocardial IRI in isolated rat hearts [27]. We observed a remarkable compensatory increase of plasma SOD activity in diabetes when reperfusion last for a longer time (from 2 hours to 3 hours), which is similar to our previous study showing that in 9 weeks of STZ-induced diabetic rats, a compensatory increase in myocardial total antioxidant capacity occurred as a consequence of the increase of cytosolic Cu/Zn-SOD, but yet it was not sufficient to prevent hyperglycemia-induced oxidative stress [34]. It should be noted that oxidative stress represents a major cause of reduced myocardial nitric oxide (NO) availability. NO, produced by enzymes called NO-synthases (NOS), has been demonstrated to play an important role in contributing to cardiovascular homeostasis and enhanced bioavailability of endogenous NO has been shown to protect the heart from myocardial IRI [35]. Physiologically, NO is mostly produced from endothelial NO-synthases (eNOS), which exhibits beneficial effects to the heart. Under pathological condition, including diabetes and ischemia stimuli, inducible NOS (iNOS) is excessively expressed and produces large amount of NO, which in the concomitant presence of excessive superoxide formation results in the formation of peroxynitrite, leading to exacerbated myocardial injury at the setting of ischemia reperfusion [30]. In the present study, reduced myocardial NO and increased nitrotyrosine were observed in diabetic rats compared to control both before and after ischemia reperfusion, which suggest that an reduction of NO bioavailability was existed in diabetic heart.

Two important signaling pathways namely, the reperfusion injury signaling kinase (RISK) pathway including PI3K/Akt signaling cascade and the protective survivor activating factor enhancement (SAFE) pathway including Jak2/STAT3 signaling cascade play key roles in enhancing myocardial NO bioavailability. Our previous reports [12,21] showed that myocardial phosphorylation of Akt and STAT3 were decreased significantly in diabetic rats at the early stage of the disease followed by decreased eNOS activation, which subsequently reduced NO production, and rendered the heart less tolerant to ischemic insult. This hyperglycemia-induced increase of IRI in diabetes can be prevented by treatment with antioxidants, N-Acetylcysteine and allopurinol, by activating both the Akt and STAT3 involved signaling pathways [12]. Activation of Akt may subsequently increase FoxO phosphorylation, resulting its inactivation via nuclear exclusion, and thus attenuate cardiac dysfunction caused by hyperglycemia [36]. Similarly, in the present study, there are reduced Akt phosphorylation at site Ser 473 and reduced STAT3 phosphorylation at site Ser727 in diabetic heart relative to controls, accompanied by reduced phosphorylation of glycogen synthase kinase (GSK)-3β at site Ser 9. GSK-3β is a downstream target of RISK signaling pathway, which is phosphorylated by Akt, and plays important roles in necrosis and apoptosis of cardiomyocytes [37]. Studies showed that activation (phosphorylation) of Akt can phosphorylate (inactivate) GSK-3β, which inactivates GSK-3β activity and confers cardioprotection [38]. However, in diabetes, phosphorylation of Akt is reduced and the activity of GSK-3β is increased, which may lead the heart damage to ischemic insult [39]. In the present study, both Akt and GSK-3β phosphorylation were reduced in diabetic heart both before and after IRI, accompanied with increased apoptotic cardiomyocytes. This indicates that impairment of activation of STAT3 and Akt may represent the fundamental mechanism responsible for the increased susceptibility of the diabetic heart to IRI.

## Conclusions

In summary, this study demonstrated that, in the early stage of STZ-induced diabetes, rats displayed increased myocardial oxidative stress and reduced antioxidant capacity together with impaired Akt/GSK-3β and STAT3 activation, which rendered the diabetic heart more sensitive to IRI. Moreover, increased myocardial injury in the current diabetic rat model depends more on the duration of ischemia but not that of reperfusion. Our findings demonstrate that the diabetic heart, even at the early stage of the disease, is more sensitive to IRI.

## Competing interests

The authors declare that they have no competing interests.

## Authors’ contributions

HL performed the study and wrote the manuscript. HL, ZL, JW, GTW, CWC, LZ and CC performed the study and/or contributed to data analysis and interpretation. MI and ZX reviewed/approved the research protocol. ZX wrote the manuscript. ZX takes full responsibility for the work as a whole, including the study design, access to data, and the decision to submit and publish the manuscript. All authors read and approved the final manuscript.
